# Theranostic mesoporous platinum nanoplatform delivers halofuginone to remodel extracellular matrix of breast cancer without systematic toxicity

**DOI:** 10.1002/btm2.10427

**Published:** 2022-10-21

**Authors:** Jie Zhang, Ziqing Xu, Yang Li, Yongzhi Hu, Jiajia Tang, Jiaqi Xu, Yafei Luo, Feiyun Wu, Xiaolian Sun, Yuxia Tang, Shouju Wang

**Affiliations:** ^1^ Laboratory of Molecular Imaging, Department of Radiology The First Affiliated Hospital of Nanjing Medical University Nanjing China; ^2^ State Key Laboratory of Natural Medicines, Key Laboratory of Drug Quality Control and Pharmacovigilance, Department of Pharmaceutical Analysis China Pharmaceutical University Nanjing China

**Keywords:** drug delivery, extracellular matrix, halofuginone, mesoporous platinum, theranostic nanoplatform

## Abstract

The enriched collagens in the extracellular matrix (ECM) of breast cancer substantially impede drug delivery. Halofuginone (HF), a potent antifibrotic agent, was effective to deplete the collagens and remodel the ECM by inhibiting the TGFβ pathway. However, the application of HF was hindered by its strong liver toxicity. Herein, mesoporous platinum (mPt) nanoparticles were constructed to load HF as theranostic nanoplatforms. mPt had a uniform spherical structure with a diameter of 79.83 ± 6.97 nm and an average pore diameter of 20 nm and exhibited good photothermal conversion efficiency of 62.4%. The obtained HF‐loaded nanoplatform (PEG@mPt‐HF) showed enhanced cytotoxicity through the combination of photothermal therapy and the anti‐TGFβ effect induced by HF. The animal imaging and histochemical assays confirmed the PEG@mPt‐HF could efficiently deliver HF to tumors (monitored by CT) and remodel the ECM by TGFβ pathway inhibition, which resulted in increased anti‐cancer efficacy. Importantly, the liver toxicity observed in HF‐treated mice was negligible in those treated by PEG@mPt‐HF. Overall, this study designed a theranostic nanoplatform to remodel the ECM with remarkably reduced systematic toxicity and enhance the therapeutic efficacy through combination treatment.

## 1 INTRODUCTION

Breast cancer (BC) is the most common cancer for women, with an incidence of 5% worldwide.^1^, ^2^ Rich extracellular matrix (ECM) is one of the tumoral microenvironment hallmarks of BC.^3^, ^4^ It is composed of enriched collagens and serves as a dynamic regulator of numerous cellular processes including cell adhesion, migration, and signaling.^5^, ^6^, ^7^ Recently, increasing findings suggested the ECM played a critical role in the therapy resistance of cancers,^8^, ^9^ because the dense structure gives rise to substantial barriers to the perfusion, diffusion, and convection of drug delivery.^10^, ^11^, ^12^ Therefore, it is a great necessity to deplete the collagen in tumor ECM to improve the delivery of drugs and thus the therapeutic efficacy.

Halofuginone (HF) is an effective antifibrotic agent whose active principle (febrifugine) was isolated from the Chinese herb Chang Shan (*Dichroa febrifuga Lour*).^13^, ^14^ HF could not only inhibit the formation of fibrosis but also deplete the collagens in cancer stroma.^15^ This antifibrotic effect was mainly from the inhibition of the transforming growth factor‐β (TGFβ) signaling pathway.^16^ TGFβ is one of the major cytokines implicated in the activation of myofibroblasts.^17^ It induced the phosphorylation of Smad3 and transformed quiescent fibroblasts to activated myofibroblasts that overexpress α‐smooth muscle actin (αSMA).^18^, ^19^ HF inhibited the TGFβ‐Smad3 signaling pathway and thus deplete the collagens in the ECM of tumors.^20^, ^21^ Our previous studies have shown that HF could enhance the therapeutic efficacy of nanoparticles by disrupting the collagens in tumor stroma.^22^ However, to visualize and optimize the dosage of HF, it is desired to load HF on nanoparticles with imaging ability, whose distribution can be readily monitored by imaging to reflect the depletion of collagens in tumor stroma. Moreover, HF provokes high toxicity to the liver and other human organs,^23^, ^24^ which further requires suitable nanocarriers to improve its biodistribution and reduce side effects.

The mesoporous platinum (mPt) nanoparticles have porous structure and high surface area, which could act as excellent drug delivery carriers. Previous reports showed that mPt would help to reduce the toxicity of chemotherapeutic drugs to normal tissue.^25^ Because of the high atomic number of Pt, mPt could be also applied as computed tomography (CT) imaging contrast.^26^ Moreover, the mPt could convert light energy to heat to exert photothermal therapy (PTT), which could be used to enhance the therapeutic efficacy of HF.

The dense collagen matrix in tumors significantly hampers the penetration and impacts the efficacy of nanotherapeutics.^21^, ^27^, ^28^ The success of PTT is directly related to the delivery of nanoparticles to tumors.^29^ Besides, the depletion of collagens would boost the immunogenic effect of PTT and thus further enhance the therapeutic efficacy.^30^ Hence, we speculate that the reduction of tumor stroma would also benefit the penetration and distribution of mPt and thus enhance its therapeutic efficacy.

Herein, we built a multi‐function nanoplatform by loading HF into polyethylene glycol (PEG) coated mPt (PEG@mPt‐HF). This nanoplatform could deplete the collagens in the stroma of BC, improve the distribution of nanoparticles, reduce the liver toxicity of HF and enhance the therapeutic efficacy by combing HF with PTT. More importantly, the success of collagen depletion could be monitored by the enhanced CT contrast due to the imaging ability of PEG@mPt‐HF.

## 2 MATERIALS AND METHODS

### 2.1 Materials

Hydrogen hexachloroplatinate (H_2_PtCl_6_, 8 wt.% in H_2_O), potassium bromide (KBr), polyoxyethylene‐polyoxypropylene ether block copolymer (F127), and 3‐(4,5‐dimethylthiazol‐2‐yl)‐2,5‐diphenyltetrazolium bromide (MTT) were purchased from Sigma‐Aldrich. Ascorbic acid (AA, purity ≥99.8) was purchased from Sinopharm Chemical Reagent Co. (Beijing, China). Thiol‐PEG‐carboxylic acid (SH‐PEG‐COOH, MW = 2000 Da) was purchased from Shanghai Bioray Biotechnology, Co. Ltd. HF was bought from Shanghai Yuanye Bio‐Technology Co., Ltd. 4′,6‐diamidino‐2‐phenylindole (DAPI) was purchased from Beyotime Institute of Biotechnology (Shanghai, China). Anti‐αSMA, anti‐CD31, anti‐TGFβ1, and anti‐Smad3 antibodies were purchased from Abcam (UK). Roswell Park Memorial Institute‐1640 Media (RPMI‐1640), heat‐inactivated fetal bovine serum (FBS), dimethyl sulfoxide (DMSO), and 0.05% trypsin–EDTA were obtained from Gibco Laboratories (NY). 4T1 cell line was bought from American Type Culture Collection (ATCC).

### 2.2 Bioinformatics analysis of BC patient

The expression profiles of collagen‐I gene (COL1A1) in pan‐cancer were displayed using Tumor Immune Estimation Resource (TIMER) database (https://cistrome.shinyapps.io/timer/). COL1A1 and COL1A2 expressions in BC were estimated on the Gene Expression Profiling Interactive Analysis 2 (GEPIA 2) database (http://gepia2.cancer-pku.cn/). To further explore the different states of collagen‐I in breast normal tissues and BC tumor tissues, the immunohistochemistry (IHC) data were mined on The Human Protein Atlas (THPA) database (https://www.proteinatlas.org/). Furthermore, their influences on BC patients' prognosis were investigated on the Kaplan–Meier plotter database (http://kmplot.com) using protein data. Besides, to investigate the relationship between collagen‐I and αSAM+ cells as well as the TGFβ pathway, the correlations of COL1A1 or COL1A2 and the marker gene of αSAM+ cells (ACTA2), also the main genes in the TGFβ pathway (TGFB1, TGFB2, and TGFB3), were explored using BC data on the TIMER database.

### 2.3 Synthesis and characterization of mPt and PEG@mPt

In the first step, mPt was synthesized. Typically, 450 mg of Pluronic F127 and 1 g KBr were mixed with 15 ml AA solution (0.1 M) and ultrasonically dispersed for 10 min. Then H_2_PtCl_6_ (0.25 ml, 0.2 M) was added to the mixture. After that, the solution was transferred into a 70°C water bath standing for 24 h. The solution turned from orange to dark, which was the color of mPt. The product was washed with deionized water (dH_2_O) three times (8500 rpm, 10 min) and finally dispersed into 7.5 ml dH_2_O for later use. To PEGylate crude mPt, 0.25 ml SH‐PEG‐COOH (2 kDa, 5 mg/ml) was added to 0.5 ml mPt solution and shaken for 4 h. Then the loading capacity of mPt was estimated. In detail, PEG (1.25 mg) and 0.5 ml various concentrations of HF (15.625, 31.25, 62.5, 250, and 500 μg/ml) were mixed simultaneously with 0.5 ml mPt solution (1 mg/ml). The products were collected by centrifugation at 8500 rpm for 10 min and washed 3 times with dH_2_O. The supernatant and wash solutions were collected to measure the ultraviolet–visible (UV–vis) absorption at 242 nm to estimate the amount of unloaded HF. The quantification of unloaded HF in the supernatant was determined from a standard curve (Figure S1). Loading capacity = (mass of total HF − mass of unloaded HF)/mass of PEG@mPt × 100%. To test the releasing profile, PEG@mPt‐HF was shaken in buffers of different pH (5 and 7.6) for 72 h. The released HF was determined by its UV–vis absorption at 242 nm after centrifugation.

Scanning electron microscopy (SEM) measurements were performed at 5 kV (Carl Zeiss's GeminiSEM 500 ultra‐high resolution scanning electron microscope from Germany). The core size of mPt was determined by a Micromeritics ASAP analyzer through the Nitrogen adsorption–desorption isotherms. Dynamic light scattering (DLS) and zeta potentials were determined by dynamic light scattering measurement Brookhaven analyzer (Brookhaven Instruments Co., Holtsville, USA). UV–vis spectra were analyzed by UV–vis‐near infrared (NIR) spectrophotometer (UV‐3600, Shimadzu). The concentration of mPt was measured by inductively coupled plasma‐optical emission spectroscopy (ICP‐OES) (Avio500, USA).

### 2.4 Assessment of the photothermal conversion efficacy and CT imaging capability

The photothermal effect of mPt was characterized in different concentrations (0, 20, 40, and 80 μg/ml) under various power of laser irradiation (808 nm, Nanjing spectrum of optics‐electrical Technology Co., Ltd.) (0.5, 1.0, and 1.5 W/cm^2^) recorded by an IR thermal camera (FOTRIC's IR camera, 220s, China). The photothermal stability of mPt was determined through 5 rounds of irradiation (5 min) flowed cooling (10 min) process. To assess the photothermal conversion efficacy, the solution of mPt (100 μg/ml) was irradiated (1 W/cm^2^) for 15 min, stable at the highest temperature, then cooled to room temperature. The following equation was used to calculate the photothermal conversion efficacy of mPt.

$$\eta =\left(\mathrm{hS}\left(T_{\max}-T_{\mathrm{amb}}\right)-Q_{0}\right)/\left(I\left(1-10^{-A}\right)\right)\times 100\%.$$

Exploring the high atomic number characteristic of platinum, the CT imaging capability of mPt was evaluated. Different concentrations of iodine and mPt (0, 200, 400, 600, and 800 μg/ml) in 1.5 ml tubes were scanned by a dual‐source CT system (Somatom Force, Siemens Healthcare, Forchheim, Germany). The CT values in 4 slices of each concentration were calculated. Parameter: the slice thickness of 0.6 mm, the voltage of 120 kVp, and the current of 23 mA.

### 2.5 MTT assay

4T1 cells in 96‐well plates were incubated with various concentrations (0, 10, 20, 40, and 80 μg/ml) of PEG@mPt or PEG@mPt‐HF for 48 h with or without the following 808 nm irradiation at 0.5 W/cm^2^ for 10 min to explore the cytotoxicity or photothermal therapeutic efficacy. The therapeutic efficacy of HF was also investigated at different concentrations (0, 31.25, 62.5, 125, and 250 nM). The cellular viability was measured by MTT assay.

### 2.6 Tumor model

To develop the tumor model, 4T1 cells (1 × 10^5^ cells suspended in 100 μl of PBS) were subcutaneously injected into the right flank of each female BALB/c nude mice (6 weeks old). When the tumors of the mice reached 100 mm^3^, the mice were used for the following experiments.

### 2.7 Tumor inhibition assay

First, the previously prepared mice were randomly divided into five groups: control with normal saline treatment (*n* = 4), HF (0.05 mg/ml) (*n* = 4), PEG@mPt (1.7 mg/ml) + laser (*n* = 4), PEG@mPt‐HF (1.7 mg/ml, equivalent to 0.05 mg/ml HF) (*n* = 4), and PEG@mPt‐HF (1.7 mg/ml, equivalent to 0.05 mg/ml HF) + laser (*n* = 4). They all received intravenous (i.v.) injections (300 μl) every 3 days for 2 weeks, receiving a total of 5 doses. The mice in the PEG@mPt + laser and PEG@mPt‐HF + laser group received 808 nm laser irradiation (0.5 W/cm^2^) for 5 min 24 h after the final injection. The temperature of tumor was monitored using an IR thermal camera. One tumor in each group was harvested for hematoxylin and eosin (H&E) staining to measure the size of necrotic area 24 h after irradiation.

### 2.8 Masson's trichrome staining and IHC analysis

To assess the amount of collagens in each group after treatment, at least three tumors in each group were sliced to stain with Masson's trichrome, αSMA, TGFβ1, Smad3, or CD31 antibodies. Five ROIs were randomly selected to quantify the amount of collagens by the Image Pro software. The levels of TGFβ1, Smad3, and αSMA expression were determined by their integrated optical densities (IODs) in five randomly selected ROIs in each slice. The average diameter of vessels was measured on the CD31‐stained slices. More than 20 vascular diameters were measured in each slice.

### 2.9 Assessing the distribution of NP in vivo

Twenty‐four hours after the mice received the last injection (*n* ≥ 4 mice/group), they were scanned by dual‐source CT system. The CT value of each tumor was calculated. Parameter: the slice thickness of 0.6 mm, the voltage of 120 kVp, and the current of 23 mA.

The mice were euthanatized after the CT scan. Then tumors and major organs (heart, liver, spleen, lung, and kidney) of mice in PEG@mPt and PEG@mPt‐HF groups were collected. All of the samples were weighed and digested to estimate the Pt concentration by ICP‐OES.

### 2.10 Toxicity assessment

First, hemolysis experiment was carried out to test the biosafety of PEG@mPt. One milliliter of blood was washed with saline until the supernatant was clear (centrifuged at 1000*g*, 4°C for 5 min). The obtained red blood cells (RBCs) were diluted with 5 ml of saline. Then, 0.2 ml of the RBCs were added to 0.8 ml of various concentrations of PEG@mPt (10, 20, 30, 40, 50, 60, 70, and 80 μg/ml) and placed in 37°C water bath. The positive and negative controls were 0.8 ml of water and saline, respectively. After 2 h, all samples were centrifuged at 1000*g*, 4°C for 5 min to collect the supernatant. Finally, the UV–vis absorptions of the supernatant at 490 nm were measured. Hemolysis ratio = (absorption of sample − absorption of negative control)/(absorption of positive control − absorption of negative control) × 100%.

The weights of mice were measured every 3 days for 24 days. In the end, the blood of mice in the five groups was investigated for blood routine and biochemistry analysis. The blood routine examination consisted of the counting of RBC, white blood cell (WBC), platelet (PLT), and hemoglobin (HGB). The biochemistry analysis included the level of alkaline phosphatase (ALP), aspartate aminotransferase (AST), creatinine (Cre), and blood urea nitrogen (BUN). Finally, the major organs (heart, liver, spleen, lung, and kidney) of all mice were obtained and stained with H&E.

### 2.11 Statistical analysis

Data are presented as means ± standard deviation of ≥3 independent experiments performed. Student *t*‐test was used for comparison between two groups. Kaplan–Meier plot was used to analyze the association of COL1A1 or COL1A2 levels with BC patient survival. Correlation between pairs of COL1A1 or COL1A2 and ACTA2, TGFB1, TGFB2, or TGFB3 in BC was analyzed using Spearman's rho test. Statistical significance (*p*) was displayed as *p < 0.05, **p < 0.01, ***p < 0.001. The IC50 values corresponding to 50% inhibition of cell viability were calculated through the four‐parameter logistic equation.

## 3 RESULTS AND DISCUSSION

### 3.1 ECM enrichment in BC patients

**FIGURE 1:**
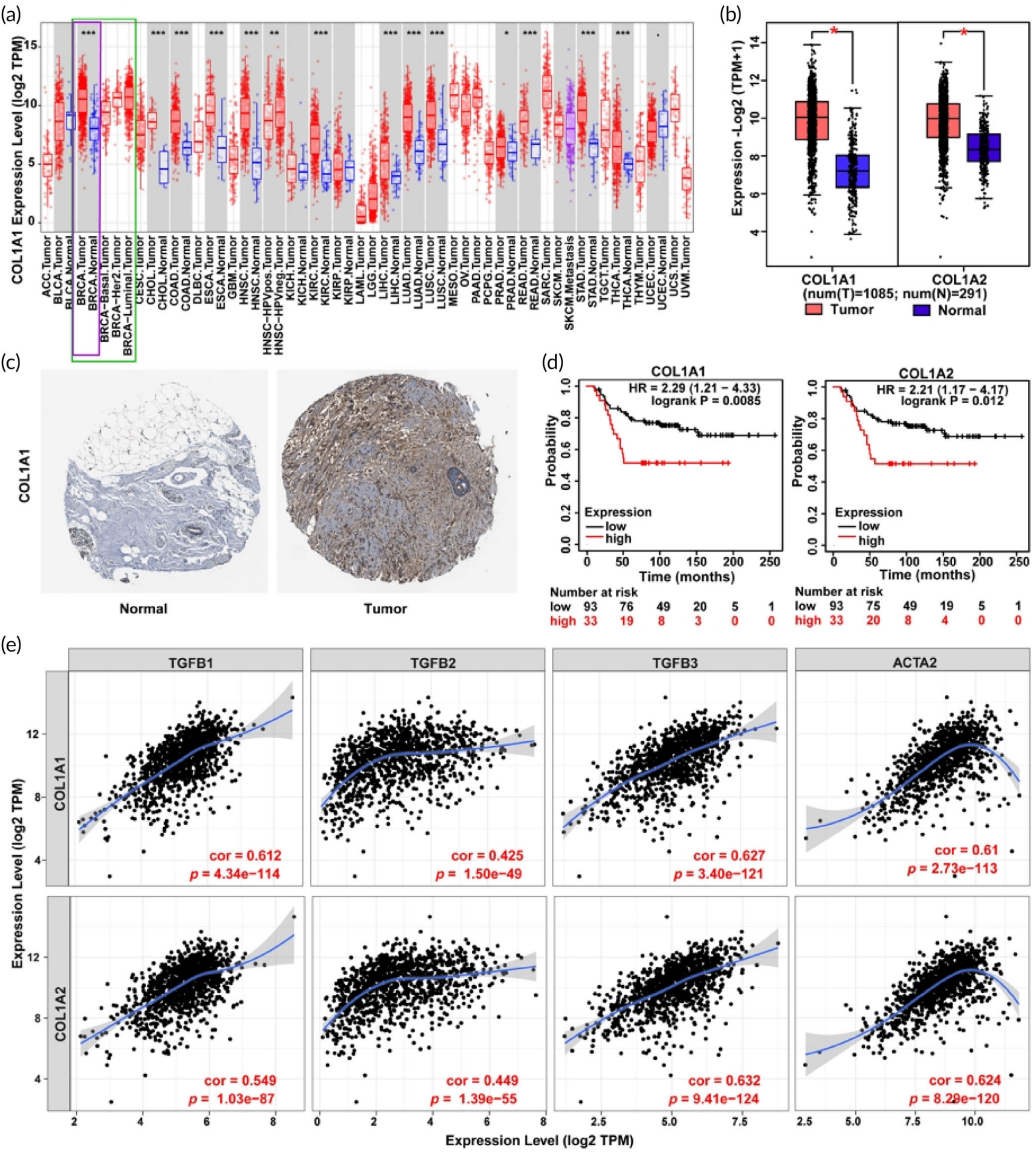
(a) Gene expression profiles of COL1A1 across TCGA/GTEx data in pan‐cancer. (b) Differential expressions of COL1A1 and COL1A2 between BC tumors and normal breast tissues. (c) IHC images with COL1A1 staining in normal breast tissue and BC tumor of patients. (d) The survival curve of patients with high and low levels of COL1A1 and COL1A2 gene expression. (e) The relationship between the marker genes of collagen‐I and the TGFβ pathway, αSAM+ cells. *p < 0.05, **p < 0.01, ***p < 0.001

As collagen‐I is the major component of ECM, its gene expression profiles across cancers were identified using TCGA/GTEx database. It is noted that collagen‐I was overexpressed across cancers, especially in BC patients (Figure 1a,b). The IHC data also confirmed that collagen‐I was highly expressed in BC tumors compared with normal breast tissue (Figure 1c). Moreover, the high expression of collagen‐I was significantly related to the poor prognosis of BC patients (Figure 1d). The expression of collagen‐I was also positively related to the expression of TGFβ and ACTA2 (corresponding to αSAM) (Figure 1e), indicating the important roles of TGFβ and αSAM+ myofibroblast in the regulation of ECM. These results showed the ECM was enriched across cancers, especially in BC. Anti‐TGFβ may remodel the ECM of BC and improve the outcome.

### 3.2 Characterization

**FIGURE 2:**
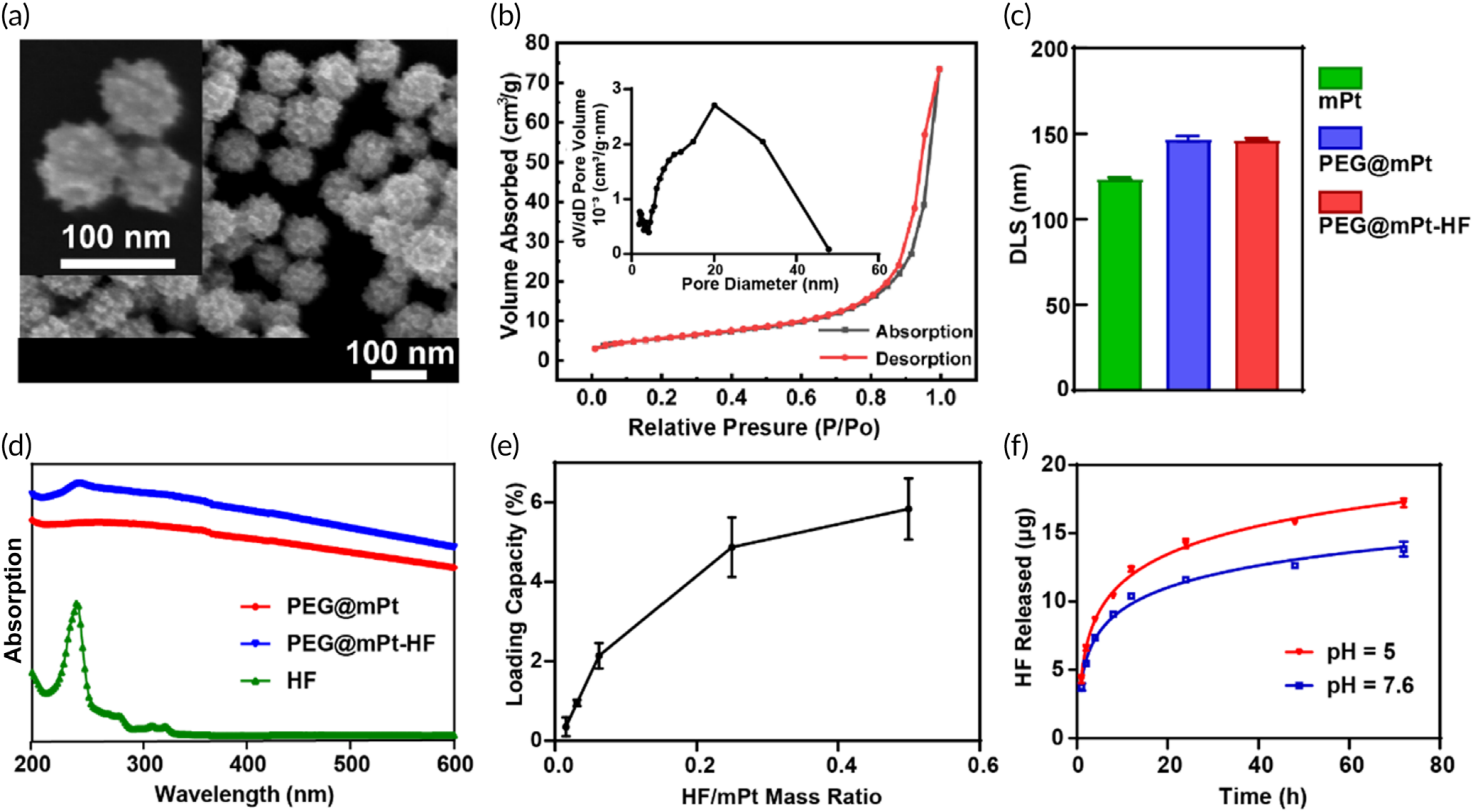
(a) SEM images of mPt (bars: 100 nm). (b) Nitrogen sorption isotherms and pore size distribution of the mPt. (c) DLS of mPt, PEG@mPt, and PEG@mPt‐HF. (d) UV–vis absorption spectrum of mPt, PEG@mPt‐HF, and HF. (e) The HF loading capacity of PEG@mPt. (f) HF release profiles from PEG@mPt‐HF at different pH

mPt nanoparticles were synthesized to load the anti‐TGFβ drug, HF, to remodel the ECM of BC. The SEM images indicated that mPt possesses uniformly distributed mesoporous structures and a well‐defined spherical shape (Figure 2a). The diameter of mPt particles in SEM images is 79.83 ± 6.97 nm. The nitrogen sorption isotherms of the mPt were further measured (Figure 2b), which showed a type IV curve, indicating a typical mesoporous structure. The average pore diameter was 20 nm and the surface area was 23 m^2^/g according to the BJH desorption algorithm. The mesoporous structure and large surface area were suited for loading HF. Next, we characterized the hydrodynamic diameters of mPt, PEG@mPt, and PEG@mPt‐HF, which were 123.86 ± 0.62, 147.24 ± 1.42, and 146.76 ± 0.76 nm respectively (Figure 2c). The diameter of the PEG@mPt was slightly larger than that of mPt, indicating PEG connected with mPt successfully. The zeta potentials of mPt, PEG@mPt, and PEG@mPt‐HF were measured to be −42.88 ± 4.28, −25.17 ± 1.10, and −48.76 ± 1.10 mV. UV–vis absorption spectrum of PEG@mPt‐HF exhibited the peak absorption of HF at 242 nm, further indicating the HF was loaded in mPt. As shown in Figure 2d, when the mass ratio reached 0.5:1 (HF:mPt), the loading capacity reached plateau at 5.83%. After incubation in pH 5 and 7.6 buffers, 47.44% and 59.03% of loaded HF were released (Figure 2f). These results showed the good loading capacity of mPt and the pH‐responsive release profile of PEG@mPt‐HF.

### 3.3 Theranostic characteristics of PEG@mPt‐HF

**FIGURE 3:**
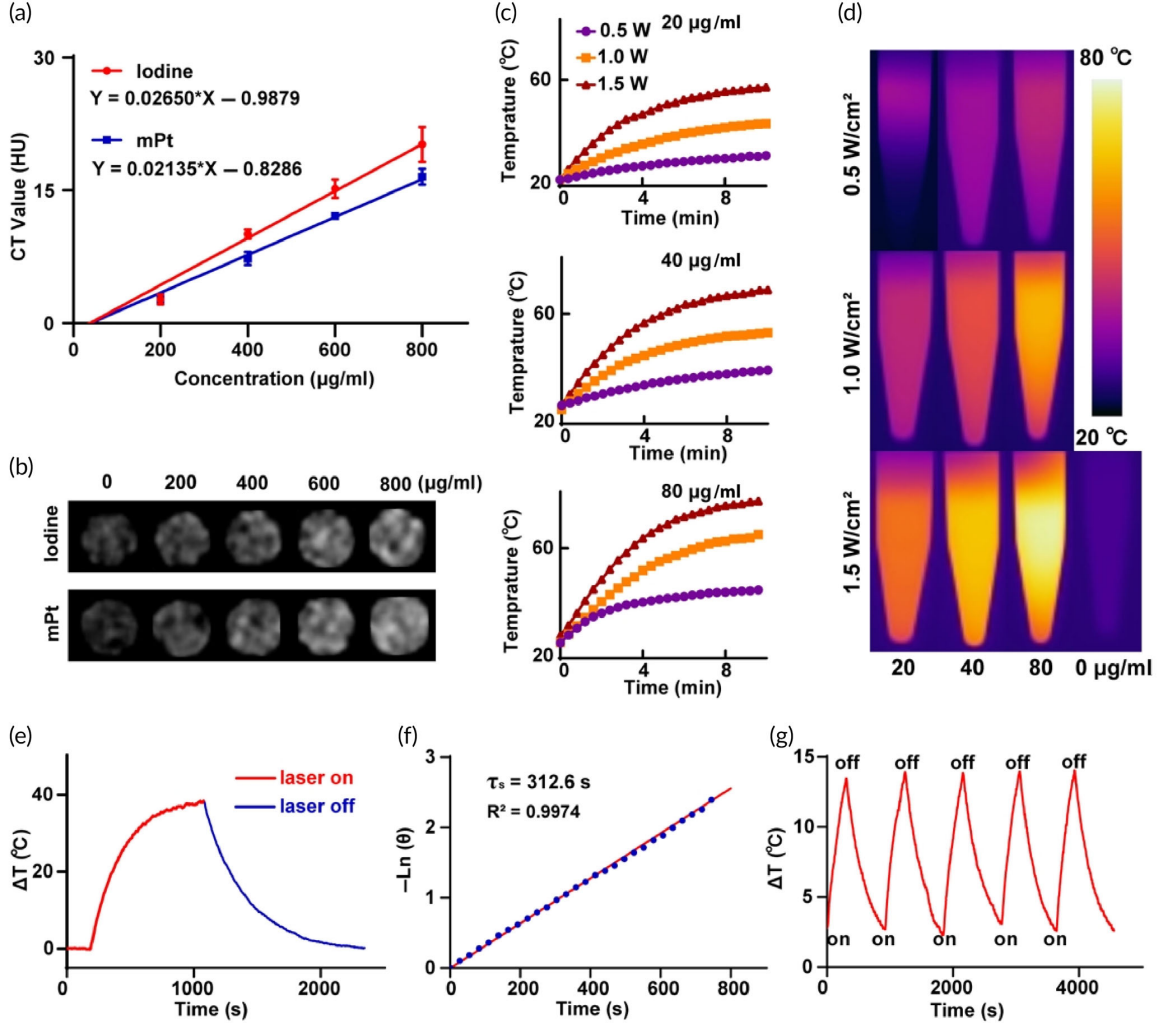
(a) The CT values of iodine or PEG@mPt (0–800 μg/ml in 1.5 ml tubes). (b) CT images of different concentrations of iodine and mPt. (c) Photothermal curves of mPt solution under laser irradiation for 10 min at different concentrations (20, 40, and 80 μg/ml) and different power densities (0.5, 1, and 1.5 W/cm^2^). (d) Thermography of mPt solutions and H_2_O. (e) Heating and cooling curves of 100 μg/ml mPt under laser irradiation (1 W/cm^2^). (f) The linear regression between cooling time and negative natural logarithm of driving force temperature. (g) Photothermal stability of mPt within five cycles of NIR laser irradiation. The laser irradiations were all 808 nm.

To explore the theranostic potential of mPt, its CT imaging ability was compared to the clinical contrast agent, iodine. It is shown that the contrast of mPt was similar to that of iodine at the same concentration (Figure 3a,b). Furthermore, upon 808 nm laser irradiation, the temperature of mPt solutions increases smoothly, and heat production capacity was positively correlated with the concentration of mPt and the laser power (Figure 3c,d). No significant change in temperature was observed for H_2_O during 1.5 W/cm^2^ laser irradiation (Figure 3d). The photothermal conversion efficacy of mPt was calculated to be 62.4% (Figure 3e,f). Besides, the photothermal curve of the mPt was stable during five cycles of laser irradiation (Figure 3g). In summary, these results showed that mPt possessed the potential to be used as a theranostic platform for CT imaging and PTT.

**FIGURE 4:**
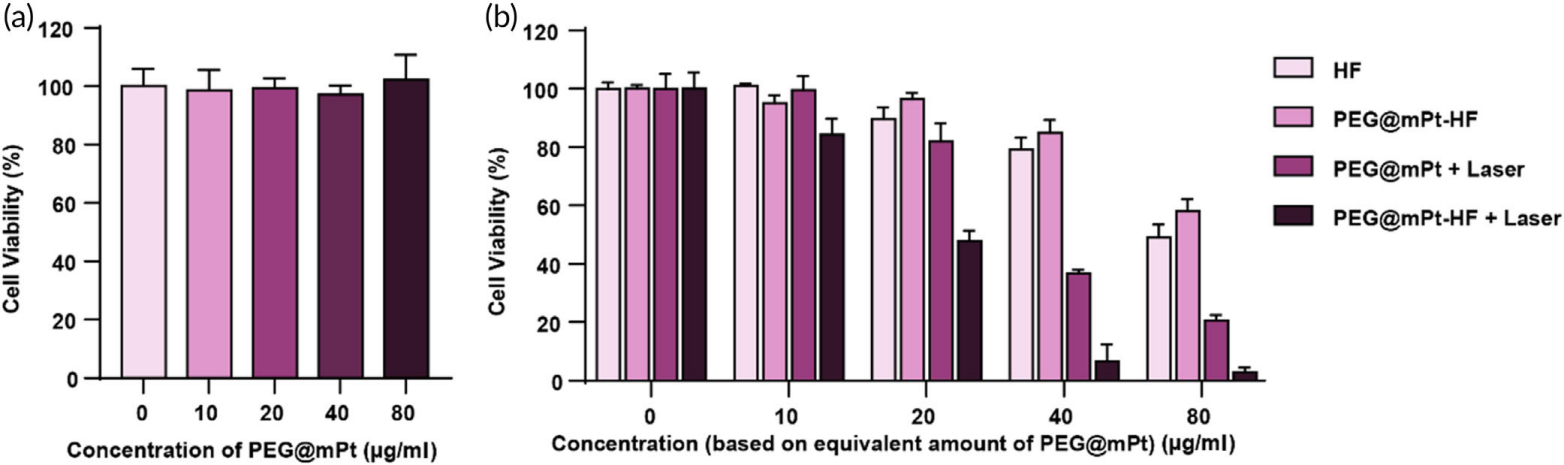
(a) The Viability of 4T1 cells incubated with PEG@mPt in different concentrations (0–80 μg/ml). (b) The viability of 4T1 cells incubated with free HF, PEG@mPt‐HF, and PEG@mPt or PEG@mPt‐HF with the following irradiation (0.5 W/cm^2^, 10 min). Each concentration had six repeats.

To investigate the anti‐cancer effect of PEG@mPt‐HF, the cell viability was tested by MTT assay. After incubation with different concentrations of PEG@mPt for 48 h, the cell viability remained above 90%, indicating the great biocompatibility of PEG@mPt (Figure 4a). As shown in Figure 4b, the PEG@mPt‐HF showed similar cytotoxicity with free HF at the same concentration, indicating the PEG@mPt could deliver the HF into the cancer cells and release it to exert TGFβ inhibition effect. It is noted that the therapeutic efficacy of PEG@mPt‐HF with laser irradiation (IC50: 20.61 μg/ml) was greater than that of PEG@mPt with laser irradiation (IC50: 27.82 μg/ml) and PEG@mPt‐HF in dark (IC50: 42.13 μg/ml). These results showed that the PEG@mPt‐HF could enhance the therapeutic efficacy of HF through the combination of TGFβ pathway inhibition and PTT.

### 3.4 ECM degradation effect

**FIGURE 5:**
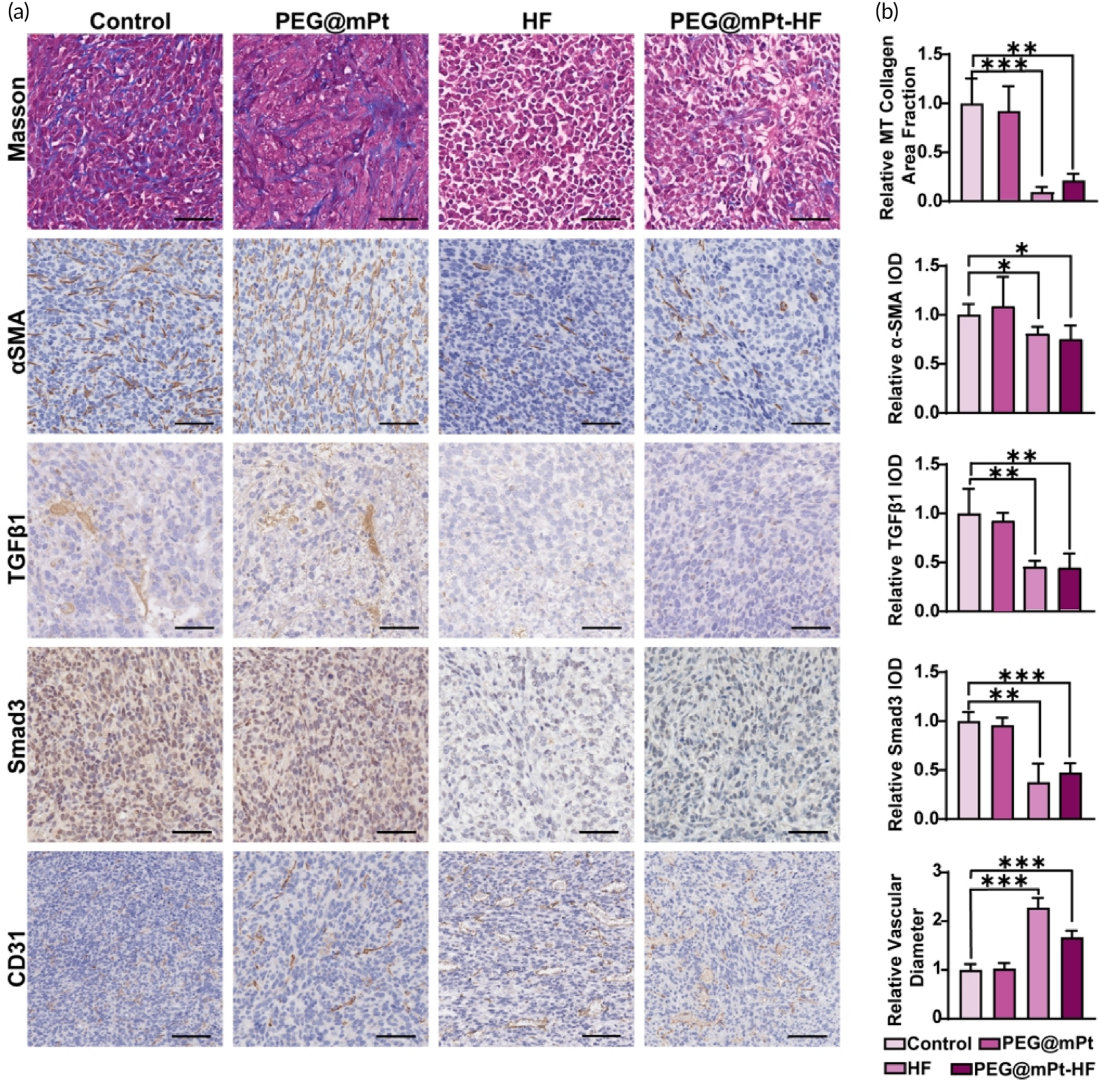
(a) Masson's trichrome stain and the IHC analysis of αSMA, TGFβ1, Smad3, and CD31 in control, PEG@mPt, HF, and PEG@mPt‐HF groups (bar: 50 μm). (b) Quantification of Masson's trichrome, TGFβ1, Smad3, and αSMA positively stained areas as well as the relative vascular diameter in different groups. *p < 0.05, **p < 0.01, ***p < 0.001

To investigate the influence of PEG@mPt‐HF on the ECM of BC, the 4T1 tumors were collected for IHC staining after i.v. injection of PEG@mPt‐HF every 3 days for 2 weeks. As shown in Figure 5, for PEG@mPt‐HF and free HF treated group, the expression of TGFβ was significantly inhibited, which results in the inactivation of myofibroblasts (αSMA+). Since activated myofibroblasts are the predominant source of collagen, the treatment of PEG@mPt‐HF and free HF reduced 78.6% and 92.2% of the collagen in the ECM of BC. It is noted the depletion of collagen resulted in dilated capillaries and intercellular space, which indicated the treatment would lower the stiffness and interstitial fluid pressure of tumors, and benefit the delivery of subsequent injected PEG@mPt‐HF.

### 3.5 CT imaging in vivo

**FIGURE 6:**
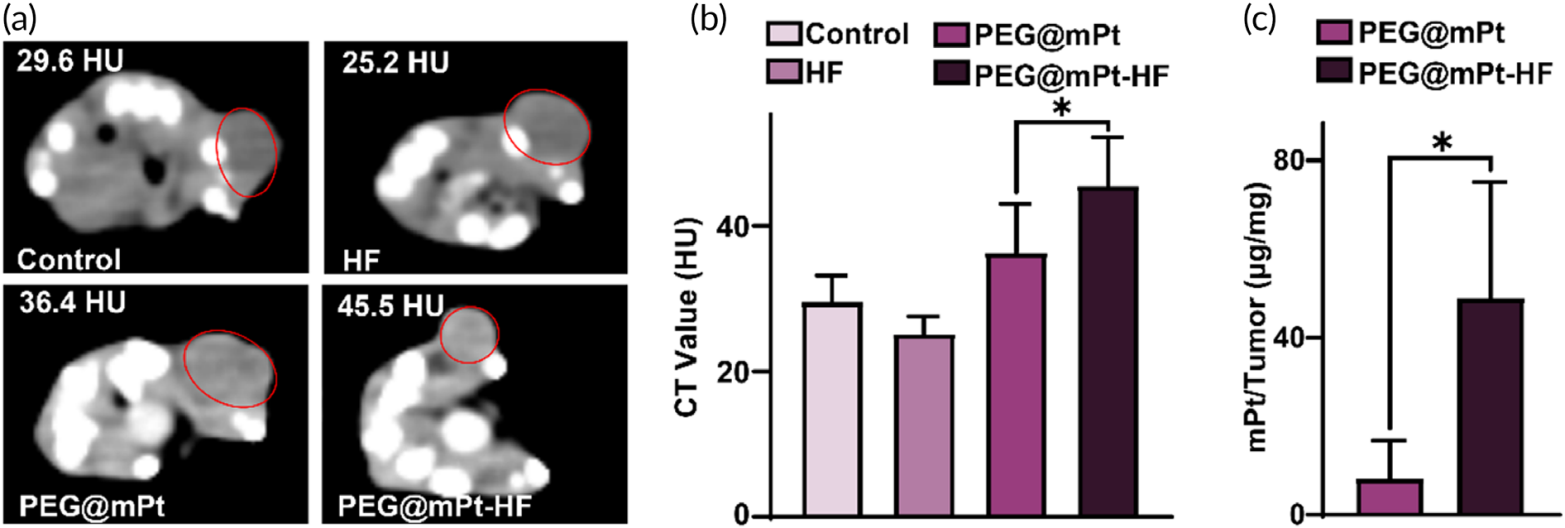
(a) The CT images of 4T1 tumor‐bearing mice after five‐times intravenous injection of saline, HF, PEG@mPt, or PEG@mPt‐HF. Red circles indicate the location of the tumors. (b) The CT values of tumor in different groups (*n* ≥ 4/group). (c) The concentration of mPt in tumors measured by ICP‐OES. *p < 0.05

To show the CT imaging ability of mPt, the mice were injected with saline, free HF, PEG@mPt, and PEG@mPt‐HF and underwent CT scan. The CT images show remarkably enhanced tumors after injection of PEG@mPt and PEG@mPt‐HF, confirming the CT imaging ability of mPt. The CT value of tumors treated by PEG@mPt‐HF (45.5 HU) was higher than that of PEG@mPt (36.4 HU) (Figure 6a,b), indicating more nanoparticles were successfully delivered to the tumors because the collagens were depleted. The distribution of the nanoparticles in the whole body was depicted in Figure S2. In line with the observation on CT, the concentration of Pt was 5.9 times higher in tumors treated by PEG@mPt‐HF than in those treated by PEG@mPt (Figure 6c). It is noted that the fold change of CT values (2.9‐fold) between PEG@mPt and PEG@mPt‐HF treated tumors was lower than that of Pt concentration, which is probably because the CT values would be more easily affected by hemorrhage and necrosis, which were common in tumors. These results indicated the PEG@mPt‐HF could improve the delivery of nanoparticles by depletion of collagens, and further enhance the CT image contrast of tumors, which is critical for the detection and assessment of BC.

### 3.6 In vivo therapeutic efficiency

**FIGURE 7:**
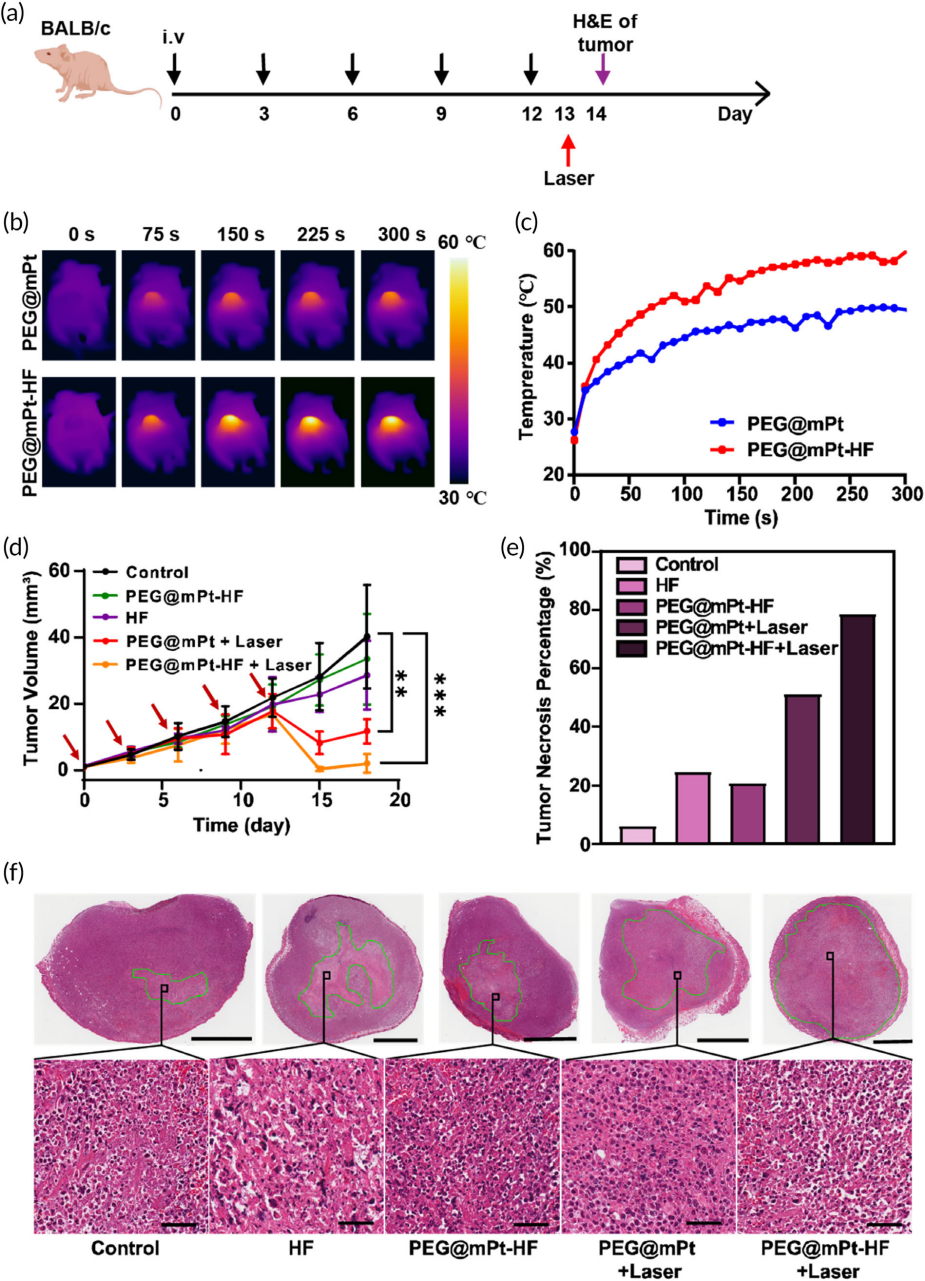
(a) The treatment timeline: Mice in different groups received 5 doses of i.v. injections. After that, mice in the PEG@mPt + laser and PEG@mPt‐HF + laser groups received 808 nm laser irradiation (0.5 W/cm^2^) for 5 min. During the period, IR thermal camera recorded the temperature changes of tumors. The next day, one tumor in each group was harvested for H&E staining. (b) Thermal images of 4T1 tumor‐bearing mice injected with PEG@mPt (upper row) or PEG@mPt‐HF (lower row) during exposure to 5 min laser irradiation. (c) The temperature curves of tumors during laser irradiation in PEG@mPt or PEG@mPt‐HF group. (d) Tumor growth curves in the five groups. (e) The tumor necrosis percentages on day 14. (f) The corresponding H&E staining of tumors in the five groups (bars of upper row are 2 mm, and bars of lower row are 50 μm). **p < 0.01, ***p < 0.001

The therapeutic efficiencies of HF, PEG@mPt‐HF, PEG@mPt + laser, and PEG@mPt‐HF + laser were evaluated on 4T1 tumor‐bearing mice. The treatment workflow was depicted in Figure 7a. Upon laser irradiation, the tumor temperature increased significantly in PEG@mPt and PEG@mPt‐HF groups (Figure 7b,c). It is noted the temperature increased faster in tumor treated by PEG@mPt‐HF, which comports with the CT imaging and ICP results. The tumor growth rates of HF and PEG@mPt‐HF groups were slightly slower but with no significant difference compared to the control group (Figure 7d), suggesting that HF alone could not inhibit the growth of tumors. Both PEG@mPt and PEG@mPt‐HF showed remarkable anti‐cancer effects after laser irradiation, but the effect was much stronger in tumors treated by PEG@mPt‐HF, implying the combination effect between TGFβ inhibition and PTT. The tumor necrosis areas observed in H&E staining slices were also evaluated 1 day after irradiation. The PEG@mPt‐HF + laser group showed the largest necrosis area percentage, which was consistent with the observed slowest tumor growth rate (Figure 7e,f).

### 3.7 Biocompatibility

**FIGURE 8:**
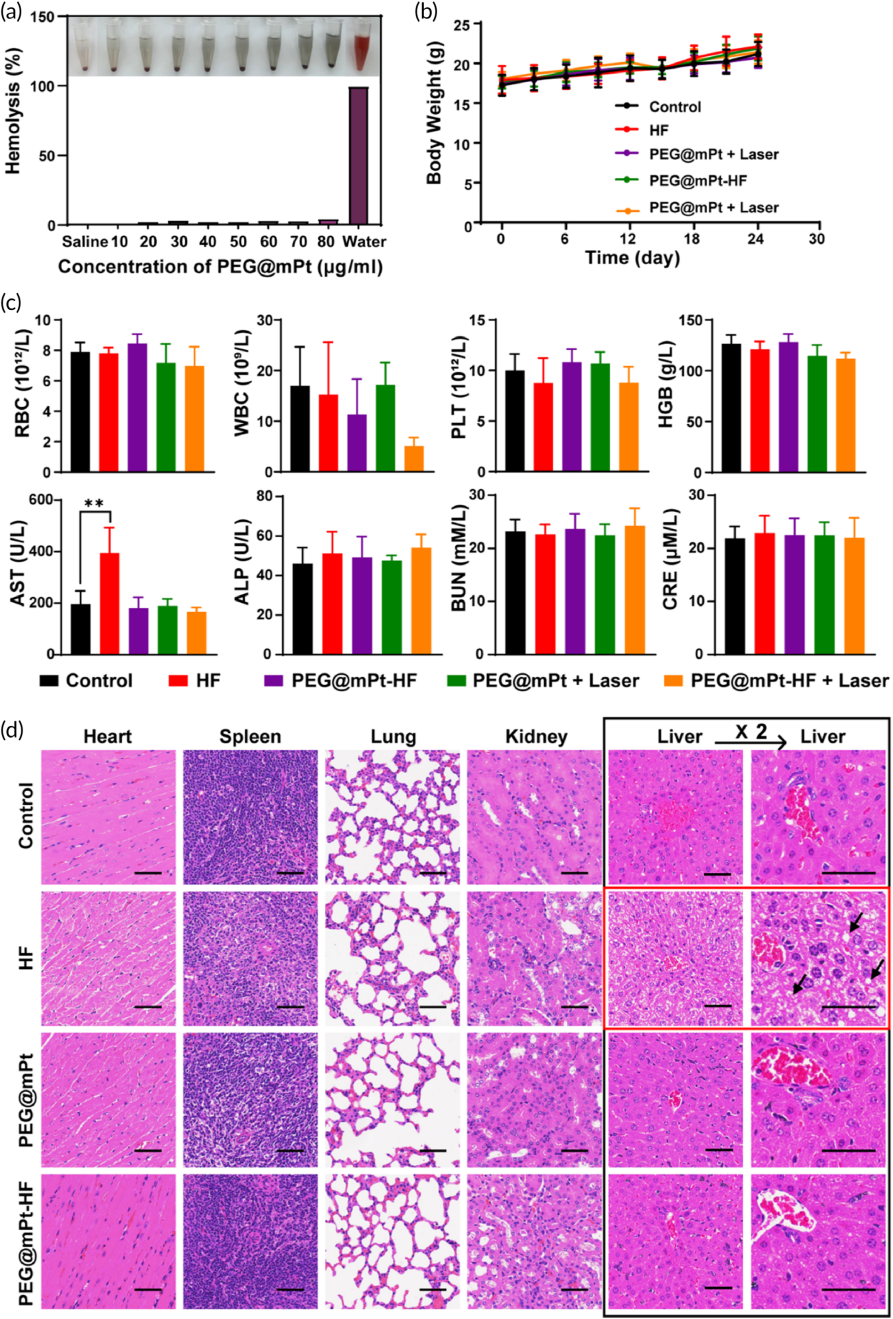
(a) Hemolysis ratio of red blood cells incubated with PEG@mPt (0–80 μg/ml). (b) The body weight of mice in different groups during treatment. (c) Blood routine data and blood biochemistry data in different groups 10 days after the completion of treatment. **p < 0.01. (d) H&E‐stained section of major organs (bar: 50 μm). As for the liver, the right column was enlarged two times by the left column. The arrows indicated the edematous and degenerated hepatocytes.

To evaluate the biosafety of PEG@mPt, various concentrations of PEG@mPt were mixed with RBCs. As shown in Figure 8a, even when PEG@mPt concentration reached 80 μg/ml, the hemolysis ratio of RBCs was less than 5%, suggesting the excellent hemocompatibility of PEG@mPt. No significant body weight changes were observed in all groups during treatment (Figure 8b). However, the hematology and serum biochemistry analyses showed free HF significantly elevated the level of AST, which is an indicator of liver damage. Remarkably, the PEG@mPt‐HF group showed normal level of AST (Figure 8c). The H&E‐stained liver sections also revealed edematous and degenerated hepatocytes in HF‐treated but not in PEG@mPt‐HF‐treated mice (Figure 8d), which comported with the result of serum biochemistry analyses. These results showed that PEG@mPt is a nanocarrier with good biocompatibility and could reduce the liver toxicity of free HF.

## 4 CONCLUSION

In summary, we built a theranostic nanoplatform by loading HF into the mPt. The PEG@mPt‐HF could exert anti‐TGFβ effect of HF to remodel the ECM of BC but minimize the hepatoxicity of HF via improved biocompatibility. The CT imaging ability of PEG@mPt‐HF made it easy to observe the drug distribution and infer the success of collagen depletion. Furthermore, the photothermal effect of PEG@mPt‐HF could enhance the therapeutic effect of HF through its TGFβ inhibition effect.

## AUTHOR CONTRIBUTIONS

**Jie Zhang:** Data curation (equal); formal analysis (lead); visualization (lead); writing – original draft (lead). **Ziqing Xu:** Data curation (equal); formal analysis (supporting); visualization (supporting); writing – original draft (supporting). **Yang Li:** Data curation (equal); formal analysis (supporting); methodology (lead); visualization (supporting); writing – original draft (supporting). **Yongzhi Hu:** Investigation (equal); methodology (supporting); resources (equal). **Jiajia Tang:** Formal analysis (supporting); methodology (equal). **Jiaqi Xu:** Investigation (equal); methodology (supporting); validation (supporting). **Yafei Luo:** Data curation (supporting); methodology (supporting); visualization (supporting). **Feiyun Wu:** Formal analysis (supporting); resources (equal); supervision (equal). **Xiaolian Sun:** Conceptualization (equal); funding acquisition (equal); writing – review and editing (equal). **Yuxia Tang:** Conceptualization (equal); funding acquisition (equal); supervision (equal); writing – review and editing (equal). **Shouju Wang:** Conceptualization (lead); funding acquisition (lead); project administration (lead); resources (equal); supervision (lead); writing – review and editing (lead).

## CONFLICT OF INTEREST

The authors declare no competing financial or non‐financial interests.

### PEER REVIEW

The peer review history for this article is available at https://publons.com/publon/10.1002/btm2.10427.

## Supporting information


**Figure S1** The standard curve of free HF absorbance at 242 nm.
**Figure S2** The distribution profile of mPt in tumor and major organs.

## Data Availability

The data that support the findings of this study are available from the corresponding author upon reasonable request.
