# Supplementary material for: Theranostic mesoporous platinum nanoplatform delivers halofuginone to remodel extracellular matrix of breast cancer without systematic toxicity
Source: Bioeng Transl Med. 2022 Oct 21;8(4):e10427. doi: 10.1002/btm2.10427 (PMC10354758; doi:10.1002/btm2.10427)
Supplement: Supplementary file 1 — Figure S1 The standard curve of free HF absorbance at 242 nm. Figure S2 The distribution profile of mPt in tumor and major organs. [file BTM2-8-e10427-s001.docx]

**Supporting Information**

Theranostic Mesoporous Platinum Nanoplatform Deliver Halofuginone to Remodel Extracellular Matrix of Breast Cancer without Systematic Toxicity

Jie Zhang^1#^, Ziqing Xv^1#^, Yang Li^1#^, Yongzhi Hu^1^, Jiajia Tang^1^, Jiaqi Xv^1^, Yafei Luo^1^, Feiyun Wu^1^, Xiaolian Sun^2^*, Yuxia Tang^1^*, Shouju Wang^1^*

1. Laboratory of Molecular Imaging, Department of Radiology, The First Affiliated Hospital of Nanjing Medical University, Nanjing, Jiangsu, China

2. State Key Laboratory of Natural Medicines, Key Laboratory of Drug Quality Control and Pharmacovigilance, Department of Pharmaceutical Analysis, China Pharmaceutical University, Nanjing, China


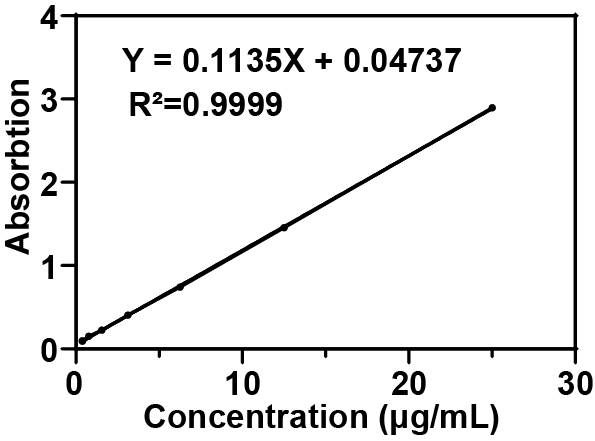


Figure S1. The standard curve of free HF absorbance at 242 nm.


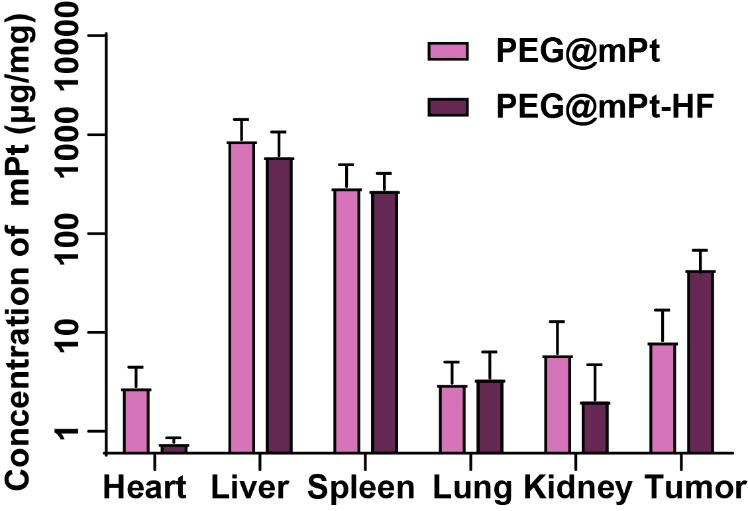


Figure S2. The distribution profile of mPt in tumor and major organs.
